# Happiness in the neonatal intensive care unit: Merits of ethnographic fieldwork

**DOI:** 10.3402/qhw.v7i0.19699

**Published:** 2012-12-12

**Authors:** Jónína Einarsdóttir

**Affiliations:** Department of Anthropology, University of Iceland, Reykjavík, Iceland

**Keywords:** ethnography, neonatal intensive care, happiness, stress, health professionals

## Abstract

Research has focused on the destructive effects of distress on professionals who work in ethically complex wards such as neonatal intensive units (NICUs). This article examines the accounts of health professionals, including nurses, pediatricians and assistant nurses, of their work at a NICU in Iceland. The aim is to understand how health professionals, who work under stressful conditions in an ethically sensitive ward, can counteract the negative sides of work too such a degree that they experience happiness. The collection of data was based on the ethnographic fieldwork, and the methods used were participant observation and semi-structured interviews. The professionals evaluated their wellbeing in line with conventional definitions of happiness. Working with children and opportunities to help others, engage in social relations and experience professional pride contributed to their happiness at work. Nonetheless, they did not dismiss the difficult experiences, and when confronted with these the professionals negotiated their meanings and the goals and priorities of work. In contrast to the findings of much quantitative and survey-based research, the professionals attributed constructive meanings to stress and argued that the positive experiences at work buffered the negative ones. Research on happiness would benefit from multifaceted methodological and theoretical perspectives. Thanks to its openness to the unforeseen, controversial, contradictory, and ambiguous aspects of human life, ethnography can contribute to happiness research and research on job satisfaction.

## Introduction

At first sight, neonatal intensive care units (NICUs) are not at all happy places. In North America and Western Europe, premature birth is currently the leading cause of permanent disability or death among infants (Baron & Rey-Casserly, 2010; Dani, Poggi, Romagnoli & Bertini, 2009; Greene, 2002; Hack, 2006). While survival rates are gradually improving, the risk of disability or other complications is high, particularly for infants of very low gestational age. However, it is difficult to estimate the likelihood of future morbidity and severity of disability (Ambalavanan et al., 2006; Bharti & Bharti, 2005; Johnson et al., 2009). Standards for estimation of health-related quality of life (HRQoL) are unclear and need further research (Mottram & Holt, 2010). Thus, it is debatable whether it is ethically justified to treat infants who may go on to become severely handicapped (Eichenwald & Stark, 2008; Janvier, Barrington, Aziz & Lantos, 2008; Kälvemark, Höglund, Hansson, Westerholm & Arnetz, 2004; Rijken, Veen & Walther, 2007; Verhagen et al., 2009). Disagreements about treatment of the least viable preterm infants are well documented, both within NICUs and between countries (Cuttini et al., 2000; Orfali, 2004).

Research on job satisfaction among health professionals working with severely sick children and ethically complex wards has focused on the destructive effects of stress and ethical dilemmas (Archibald, 2006; Braithwaite, 2008; Janvier et al., 2008; Shugerman et al., 2001). However, Lutzen, Cronqvist, Magnusson and Andersson (2003) point out that the main emphasis in such research tends to be on the negative effects of stress. They maintain that researchers should pay more attention to lived experience and be aware that stress can also be a positive factor contributing to feelings of accomplishment and pride among professionals. This article will highlight such experiences. The ethnographic data presented is derived from the research project *Abnormal birth: ethical questions and daily life*, which was initiated in 2001. The research project aimed to examine how professionals and parents deal with the birth of infants with a birth weight of 1000 grams or less, and in particular ethical questions related to their treatment and eventual end-of-life decisions (Einarsdóttir, 2006, 2007, 2008, 2009). The participants, parents, family members, and professionals at NICU were recruited through prospective and retrospective groups of prematurely born infants. The infants belonging to the prospective group were born from September 1, 2001, to August 31, 2002, and the retrospective group included infants born from September 1, 1998, to August 31, 2001. Fieldwork was conducted in the NICU from September 1, 2001 until January 2003, when the last infant belonging to the prospective group was discharged from the unit. Because it was difficult to recruit qualified nurses during the period of fieldwork, the unit was often understaffed, thus the professionals often had to deal with hectic and stressful conditions at work, in addition to the ethically sensitive dilemmas (Einarsdóttir, 2009). Nonetheless, staff members repeatedly reported moments of happiness, and consistently argued that they enjoyed their work. In this article, I will examine the narratives of health professionals, including nurses, pediatricians, and assistant nurses, about their work in the NICU with a focus on the context in which they experience happiness at work.

### Aim of study

The aim is to understand how health professionals, who work under stressful conditions in an ethically sensitive ward, can counteract the negative sides of work too such a degree that they experience happiness at work.

## Methods

Social sciences contain diverse understandings and definitions of happiness. However, happiness is most commonly treated as analogous to subjective well-being or the individuals’ estimation of their own quality of life and the associated emotions (Martin 2008; Uchida, Norasakkunkit & Kitayama, 2004, p. 223; Veenhoven, 2010). How is it possible to measure happiness? Veenhoven (2006) notes that identification of psychological correlates with a happy life has been problematic, and it is only possible to estimate happiness to a small degree by observing behavior. Therefore, Veenhoven argues, the most common reliable methodological approach is simply to ask people, either directly or indirectly, whether they are happy and to what degree. Veenhoven (2010) maintains that happiness as life-satisfaction can be measured with direct questioning and thereafter is compared across individuals and societies. The anthropologists Mathews and Izquierdo (2009a), who also treat happiness as synonymous with well-being, maintain that although happiness depends on individuals and the cultural context, its manifestations have similarities “due to our common humanity and interrelatedness over space and time” (p. 5). They maintain it is important for anthropology to understand happiness in specific societies, not only to understand its importance locally, but also for universal understanding of the human being. In contrast to the reliance on survey-based quantitative methodology that characterizes conventional happiness research, Mathews and Izquierdo (2009b) argue that “[o]nly a detailed, on-the-ground ethnography can provide the social and cultural context without which well-being in a given society cannot be fully understood” (p. 250).

The aim of the research project *Abnormal birth: ethical questions and daily life* was not to study happiness *per se*. Nevertheless the professionals’ narratives included abundant accounts about the happy hours at work. The collection of data for the project was based on the ethnographic tradition, which has been the central methodological approach for anthropology since early last century (Bernard, 2006; Crang & Cook, 2007; Hammersley & Atkinson, 2007). As pointed out by Crang and Cook (2007), “ethnographic research has developed out of a concern to understand the world-views and ways of life of actual people in the contexts of their everyday lived experiences” (p. 37). Ethnographic fieldwork may rely on a wide range of methods, though participant observation and various forms of interviewing are the most applied ones.

### Collection of data

The data on which this article rests was collected with participant observation and semi-structured interviews. Participant observation is frequently seen as the basis of anthropological methodology through which the researcher becomes familiar with people and they become comfortable with her or his presence, and simultaneously the researcher observes ongoing events and activities, as well as the environment (Crang & Cook, 2007). In the NICU, participant observation included informal chatting with the parents and staff on duty and observation of their activities and interaction with the infants and each other, as well as the particular milieu. Data were also collected through semi-structured interviews, which means that predefined list of issues guided the conversation that otherwise could have included other themes of interest. Due to heavy workload during the day, interviews were often conducted during night shifts. During the night shifts the atmosphere was most often relaxed which also allowed more time for coffee breaks, informal discussions, and engagement with the infants. Of the 50–60 professionals working at the NICU, a total of 40 individuals, including pediatricians, nurses, and nurse assistants, were interviewed. All of the pediatricians and one of the nurses were males. The interviews were characterized by informal conversation and their duration varied greatly, from ca. 45 min to roughly 2 h. The interviews, which were taped, included questions about the choice of profession, daily routines of work, and what were the most positive and negative aspects of working in the unit.

### The analysis of data

Various approaches are available to identify themes in qualitative data (Braun & Clarke, 2006). Ryan and Bernard (2003) describe 12 such procedures originating from different theoretical perspectives within the social sciences. For this article, the field-notes for observations and informal conversations as well as the transcripts of the interviews were analysed with an approach outlined by Crang and Cook (2007) “using ideas of ‘grounded theory’” (p. 133). Crang and Cook view creativity as crucial for the analysis of data, however in combination with rigorous coding and systematic way of analyzing the written texts. Thus, the written text was repeatedly read and manually coded, the first step being “open coding” in which the meaning of each statement or part of the text is scrutinized with an open mind for whatever unexpected findings (pp. 134–146). For each code identified a corresponding text was either marked or attached. The codes were listed and sorted, some were combined, others became subcategories and still others were excluded. The whole procedure is time-consuming, characterized by repeated reading, coding, and ordering of the text. At the same time, insights and gut-feelings were registered in so-called theoretical notes. The final stage was the making sense one, which is a kind of “iterative process” through which the researcher rechecks eventual misfits and follows up on hunches (Crang & Cook, 2007, p. 142).

The Findings are sorted into sub-chapters in line with the themes identified and the context that contributed to the professionals’ happiness, despite stress and ethically difficult experiences. The following sub-titles are listed: For the love of children, Doing good (the importance to help others, be useful, and be able to engage in positive social relations), Professional pride (partly through dealing with stress), and Ethical dilemmas (difficult events are outlined, nonetheless happiness exceeds hardship).

### Ethical considerations

Ethical approval of the research was obtained from The Icelandic Data Protection Authority and the Ethic Committee of National University Hospital in Reykjavik (Erindi 63/2001). Informed consent was received from each participating individual prior to interviewing and oral and written information was given about the purpose of research.

## The findings

### For the love of children

During fieldwork it became evident that “liking of children”—in Icelandic, *að vera mikið fyrir börn*^1^—characterized the professionals who worked in the NICU. When I asked one nurse why she was so enthusiastic and happy about her work, she replied, “The children are the best.” Most of the nurses and nurse assistants said they wanted to work with children because they loved children. The physicians did not describe themselves as “somebody who loved children” in the same spirit to the same extent as the nurses and nurse assistants did. Nonetheless, it was evident that many of them had, as one pediatrician said, chosen their profession “because of the children.” Most staff members realized that they wanted to specialize in intensive neonatal care when they worked in such a unit during their studies. Some believed that they had been influenced by their own experience of being admitted to a hospital as a child or having had their own child hospitalized. Many felt they had realized right from their first days of working at the NICU that this was the right place for them. When I suggested that the prematurely born infants were not able to express themselves so much, one staff member responded that, while this may be true, but also that:

You can feel their personality, you sense their personality when you are working with them … even when you work with the smallest ones. Of course, they have an unusual way of expressing themselves, but you learn that each and every one has its own personality, own temper, and are whole persons.

The professionals were consistently concerned with the individual personal character of the preterm infants, as well as their progress. One nurse explained how satisfying it was “to see the little ones and see how fast they get stronger and grow and become children. They are only fetuses to begin with—the smallest ones—but they show their character early on and become personalities.” Another nurse loved “seeing the preterm infants become people.” The professionals talked about the preterm infants as individuals and regularly referred to them by their first names. When the infants were admitted to the unit, their parents were asked for their names; this is contrary to the Icelandic tradition of keeping a newborn's name secret until the child is baptized. Technical terms such as a “case” or “patient” were rarely used.

Staff members (both male and female), often spoke to the infants in a low and gentle voice. They addressed them by their name, asked about their well-being, and explained what was going on. They sometimes also commented on the infants’ physical development or progress. The nurses and assistant nurses, who spent most of their time attending to the infants, sometimes talked about them as “my children.” As one nurse explained, “It is so much fun watching the preterm infants—later they visit you and you feel that you are part of their lives. You helped them survive. You think: this is my child.” Another nurse said that not only was the job exciting but “You also get to have about 300 children every year.”

### Doing good

One of the factors that contributed to happiness at the NICU was the feeling that the work had rapid and positive results, which could be physically visible or expressed in numbers. The staff members underlined that the treatment and care they offered to the preterm infants produced quick results, which was not always the case with other patients. One nurse explained: “What is so positive about working with preterm infants is that they get better, even the smallest ones, and I feel that I have a great deal of control over it.”

An NICU is a challenging workplace and staff members argued that those who work there need certain qualities. One crucial quality was the ability to interact with parents. As one nurse said:

I like the interaction with the parents … I enjoy interacting with people. I feel that you are so involved from the beginning and until they [the preterm infants] are discharged from the hospital. This really suits me.

It was important to care for the parents as well as their infants, and not all parents were easy to get along with. “The parents are often worried—but they can usually be calmed when everything is going well,” a nurse explained, underlining that it was especially enjoyable to work with parents when they were grateful, and “they are grateful for everything—for the technology and the knowledge.”

Regardless of which category they were part of, staff members mentioned that it was important to be useful—to have a good influence and to help others. One said, “When I feel useful and help parents, my work becomes a source of pleasure.” At first, the parents were shocked upon entering the emergency ward and seeing all the tools and tubes to which their infant was attached. The emergency ward was crowded with incubators and had a particular smell. Despite attempts to keep the ward dark and quite, the monitors blinked and alarm beeps regularly broke the silence. Staff members, especially nurses, were keen to teach parents how to manage the technical equipment and interpret their measurements. One nurse said that she enjoyed “when something positive is happening. There is always something to be happy about—even when the children start to suck, or have a bath.” The moments of happiness occurred when parents were instructed on how to feed their infant, change the diaper, or wash their infant inside the incubator. However, the greatest moment of all was to help parents take their infant out of the incubator for the first time. Such moments were a sign of progress, both for the staff and parents.

The atmosphere in the ward for infants who were awaiting discharge was more relaxed than in the emergency ward, and the staff referred to it as the “body-building ward” (Icelandic: *vaxtaræktin*). Feeding the tiny infants, which was the task of the nurse assistants and parents, could be time-consuming but was also rewarding. At times, the assistant nurses would refer to particular infants as “heavy drinkers,” while others were “lazy.” Professionals and parents both followed the infants’ achievements in gained weight closely, as this is a crucial indicator for discharge. After discharge, the children had a follow-up scheme with the physicians, who said how much they enjoyed meeting the infants again and seeing how they had fared. Parents sometimes sent photographs of their children to the NICU or passed by with their child, which the staff appreciated.

### Professional pride

Beyond the capacity to engage in personal relations and love for children, the staff agreed that the work in the NICU required the ability to deal with stress, in addition to thorough professional knowledge. The work was primarily characterized by great variation in workload and stress. Therefore, it was crucial to be able to deal with a crisis, particularly for the physicians and the nurses. No two days were the same, and it suited some to work “in action and then relax in between.” As one physician explained,

The extremes are there. You have an extremely sick preterm infant and the trick is to save its life, followed with the associated drama, and usually it all goes well.

A nurse maintained that “people are either able to work in such a place, in such an environment, or they just don't thrive there.” She continued, saying, “There is stress … you need a certain amount of stress but it can go too far … partly you're a stress addict.” Another nurse said that this work was her dream job; being “a stress addict” suited her since “it's a kind of adrenalin.” Another nurse said that the work fitted her personality, since “being a calm person, I work well under stress,” and right from the beginning she “got stuck into the job.”

All of the professionals agreed that up-to-date knowledge and competence was of paramount importance, and that it was rewarding to work with others who had high professional aspirations. Professional skills were essential: “You must know what you are doing.” It was necessary to master the latest scientific advances in a profession characterized by rapid change and development. A nurse explained that it was rewarding to hear collaborators say “ask X—she knows,” but also “when you know you know what you are doing, and the parents trust you.” The ultimate proof of professionalism was reflected in statistics. The physicians highlighted that preterm birth was the main cause of child death, and that Iceland had experienced extremely low child mortality; in some years it had been the lowest in the world.

Staff members shared their experiences with each other, which created a sense of community. They were not allowed to talk about individual patients outside the workplace, so it was important to have someone at work to discuss with. As one nurse explained, this was especially important when “things are not going so well with certain children and you see a moral dilemma approaching.” “Here we understand each other,” said another. This nurse added that, although she liked all the staff, “there are some with whom you have more in common with than others, and of course … there is whispering in corners and things like that.” Indeed, disagreements about certain issues were inevitable, often regarding ethical dilemmas.

### Ethical dilemmas

Within the NICU, there was a constant concern about the risk of prematurely born infants becoming severely disabled when “over-treated.” Unnecessary suffering of the infant was also an issue that caused some discomfort, particularly for the nurses. Within the NICU, it was accepted that the physicians often disagreed on ethical issues. In contrast, the nurses were generally seen as a coherent pragmatic group who were critical of excessive treatment. However, in private, there was a great deal of disagreement on ethical issues within all the professional groups. For instance, views on the concept of quality of life varied greatly. Some staff argued that they were not able to judge what life was worth living, while others felt it was hypocritical not to admit that “everybody wanted to have a healthy child.” Some disapproved of “hopeless treatment” that might save a life not worth living, while others stressed the importance of never giving up hope for a cure or that all infants had the same right to life, regardless of their disability. A nurse assistant was satisfied giving good care to infants “just as they are.” Another nurse argued that “It is very important that the result is a good one. One should not deal with things that greatly disappoint people, to get a damaged child.” The role of the parents in end-of-life decision was also an area of disagreement. The varying opinions on ethical issues were articulated with reference to individual life experiences, common sense, human rights, religion, and human nature.

Periods of uncertainty and imminent death were the most difficult ones. All staff members admitted that they found it emotionally difficult when an infant died. Witnessing parents in grief was always heartbreaking. However, considering the condition of some extremely prematurely born infants, death is sometimes seen as inevitable. Some found the death of infants to always be unjust, while others maintained that death sometimes alleviated unnecessary suffering. Some professionals argued that survival was not always the ultimate aim of their work. Only rarely did someone justify death with reference to religion, destiny, or a higher cause. However, many pointed out that it was important to look at the total picture to understand what it was like to work in a NICU. “The hardest moments are when a child dies or when things go badly,” a nurse explained, adding that she did find her job enjoyable: “I guess it's the little ones who are so exciting, those 1000–1500 [grams] and having just entered a crib.” She was referring to the infants who had survived the most dangerous period and were getting closer to be discharged. One should remember that “most of them go home with a smile … whether they have been here for two days, 10 days or 10 months.” This particular nurse's favorite work was discharging the infants.

Most of the staff emphasized that they enjoyed the work, even though it was hard at times. Nobody mentioned material factors, such as salaries, as a reason for their satisfaction, and many pointed out that the working conditions and hours of work were not optimal. Of course, there were moments of sorrow, and “sometimes everything seemed difficult.” Still, working at the NICU was enjoyable because there were “more miracles, positive things that happen so the negative aspects don't suffocate you.” One nurse explained that she was often asked how she could work in such a place where infants died. She explained that her answer was that the happy moments at the unit truly outnumbered the sad ones. Many other staff members also said that they experienced more happiness at work than hardship.

## Discussions

The NICU professionals who participated in the current study accounted for their daily routines at work as well as extraordinary events. The aspects that contributed to their satisfaction—love for children, successful engagement in social relations, helping others, professional pride, and working with individuals with high professional aspirations—are well documented happiness variables (Bekhet, Zauszniewski & Nakhla, 2008; Borgonovi, 2008; Grant & Sonnentag, 2010; Schiffrin & Nelson, 2010). Simultaneously, the professionals recognized periods of heavy workload, stress, emotionally difficult experiences and conflicts, which is in line with findings that happiness and hardship can coexist (Dunn, Uswatte & Elliott, 2009; Folkman, 2008; Powdthavee, 2007, 2010; Veenhoven, 2006). The accounts also reveal how the professionals attributed positive meaning to heavy workload and stress through which they experienced excitement and the opportunity to demonstrate their competence. In contrast, studies abound that confirm the negative effects that stress has on job satisfaction among health professionals and not least those working with severely or terminally ill patients (Applebaum, Fowler, Fiedler, Osinubi & Robson, 2010; Archibald, 2006; Braithwaite, 2008; Epstein, 2010; Georges & Grypdonck, 2002; Hayes et al., 2006; Kälvemark et al., 2004; Lu, While & Barriball, 2005; Utriainen & Kynga, 2009).

Aiming to understand how humans transfer difficult experiences into positive emotions through meaning-making, coping research ought to be a way forward. Coping has conventionally been defined as “thoughts and behaviors that people use to manage the internal and external demands of situations that are appraised as stressful” (Folkman & Moskowitz, 2004, pp. 746–747). Numerous types of coping have been identified, including the meaning-focused coping that occurs when an individual, through reference to beliefs, values, or existential goals, manages “to motivate and sustain coping and well-being during a difficult time” (Folkman, 2008, p. 7). Sub-categories of meaning-focused coping have been identified such as benefit finding and reminding, setting new goals, reordering of priorities, and attributing adverse events with positive meaning. Folkman (2009) calls for new approaches within meaning-making research that allow rigorous measurement of the “ways in which people access positive meaning during the coping process” (pp. 75–76). She acknowledges that carefully collected qualitative data can provide “rich descriptions of meaning” but its analysis “is a labor-intensive process” (p. 75). On that ground Folkman seemingly rejects qualitative approaches. Park (2010, p. 293) concludes that it is a priority to “understand what meaning making is and then ask for whom, and under what circumstances, are particular types of meaning-making and meaning made helpful and why?” She also highlights the importance of the social and cultural aspects of meaning-making, because meaning-making “occurs not only intraphysically but also interpersonally” (p. 292). Park underlines the importance of methodological improvements and urges researchers “to go beyond self-reports when possible” (p. 291).

The proponents of the meaning-focused coping research apparently strive to construct both objective and culturally sensitive measures of subjective human experience, preferably without giving voice to the actors through self-reports or qualitative data. In contrast, I argue that the professionals’ accounts of their lived experience presented in this article highlight the merits of the ethnographic methodological approach and qualitative data. The aforementioned subcategories already identified by the meaning-making research can easily be identified in the professionals’ accounts presented in this article. The professionals did not dismiss or downgrade the emotionally and ethically difficult aspects of their work, or their hard workload and stress. Instead, they evaluated their well-being at work as a whole. Not only did they engage in meaning-making that might have reduced their negative emotions, but they also argued, in the spirit of Bentham, that when their overall working situation was taken into account, happiness exceeded hardship, which made hardship bearable. Ultimately, the professionals argued that their personality and individual characteristics, their devotion to children, social competence, professional ambitions, and stress tolerance contributed to their well-being in a stressful and ethically complex setting like the NICU.

The research on positive stress has a long history (Bicknell & Liefooghe, 2010). Nonetheless, researchers studying distress in ethically sensitive health care settings tend to focus exclusively on its negative aspects. The findings presented here indicate that the processes through which positive experiences can buffer or counteract the difficult ones are worthy of further research. That research should consider the ethnographic approach and take notice of McCarthy and Deady (2008) who argue for a multidisciplinary approach.

## Conclusion

The aim of this article, which is based on data collected with an ethnographic approach among professionals in neonatal intensive care in Iceland, is to understand how health professionals managed to experience happiness despite otherwise often difficult conditions at work. The professionals who participated in the research evaluated their well-being in line with conventional definitions of happiness, without ignoring the difficult sides of their work. The circumstances that contributed to their happiness were partly explained with individual personality, such as their liking of children and competence to engage in social relations, and partly with the specificity of neonatal intensive care that offered opportunities to help others and experience professional pride. In line with the meaning-focused coping research, when confronted with adverse experiences the professionals negotiated their meanings as well as the goals and priorities of their work. Ultimately, they argued that the happiness they experienced at work buffered or counterbalanced the negative aspects.

The results of this study highlight the merits of the ethnographic approach for research on job satisfaction and happiness. First, although the study did not set out to focus on happiness, a large number of positive memories emerged in the field notes as an important part of the professionals’ lived experiences. Second, unlike much of the existent research, stress was identified as a factor that contributed to professional pride and job satisfaction. A methodological focus on singular conditions such as ethical uncertainty and distress may obscure the understanding of the general working conditions of neonatal professionals, or those working in similar settings, and may result in an overly negative evaluation of their lived experiences. While not all types of stress contribute to happiness, meaningful stress or challenges do. This study also suggests that positive experiences balance out the negative ones. The final lesson is that research into happiness would benefit from multifaceted methodological and theoretical perspectives. An ethnographic approach has much to offer, not least due to its openness for and acceptance of the unforeseen, controversial, contradictory, and ambiguous aspects of human life.

## Notes

1 Daily Icelandic language makes no distinction between infants and children. Therefore, the word “children” is used in some of the direct citations even when the children in question are newborns.
